# Case Report: Multidisciplinary approach for complete resection of primary advanced low-grade serous ovarian carcinoma involving the iliac vessels and paraspinal region

**DOI:** 10.3389/fonc.2026.1762009

**Published:** 2026-02-25

**Authors:** Naoyuki Ida, Shoji Nagao, Atsushi Fujikawa, Yui Tanaka, Momoko Tanioka, Ryoko Imatani, Yoshinori Tani, Hanako Sugihara, Hirofumi Matsuoka, Kazuhiro Okamoto, Junko Haraga, Chikako Ogawa, Keiichiro Nakamura, Hisashi Masuyama

**Affiliations:** Department of Obstetrics and Gynecology, Okayama University Graduate School of Medicine, Dentistry and Pharmaceutical Sciences, Okayama, Japan

**Keywords:** anterolateral thigh flap, extra-pelvic invasion, gynecologic oncology, low-grade serous ovarian carcinoma, multidisciplinary surgery, surgical limit

## Abstract

**Background:**

Low-grade serous ovarian carcinoma (LGSC) is characterized by indolent progression and relative resistance to cytotoxic chemotherapy, making complete cytoreduction the key prognostic determinant. However, extra-pelvic invasion presents significant surgical and functional challenges requiring coordinated multidisciplinary management.

**Case presentation:**

A 62-year-old woman with FIGO stage IVB LGSC presented with right inguinal swelling infiltrating the femoral vein and abdominal wall. MRI and PET-CT revealed bilateral ovarian tumors and multiple lymph node metastases. A multidisciplinary operation involving gynecologic, orthopedic, plastic, and gastrointestinal surgeons was conducted. The procedures included total abdominal hysterectomy, bilateral salpingo-oophorectomy, omentectomy, pelvic and para-aortic lymphadenectomy, en bloc resection of the right inguinal lesion, femoral vein repair, and anterolateral thigh flap reconstruction. Complete resection (R0) was achieved. Postoperative recovery was favorable, with transient leg edema resolving within 4 months. The patient remains disease-free at 13 months after surgery.

**Discussion:**

Strategic multidisciplinary collaboration enabled complete resection and functional preservation in this chemotherapy-resistant LGSC case. We propose the “Four Surgical Limits” framework—anatomical, oncological, functional, and interdisciplinary—as a structured concept guiding operative decision-making beyond conventional boundaries.

**Conclusion:**

Multidisciplinary collaboration can overcome traditional surgical and oncologic barriers, achieving both radicality and quality-of-life preservation in advanced LGSC.

## Introduction

Low-grade serous ovarian carcinoma (LGSC) accounts for approximately 2% of epithelial ovarian cancers (1). It exhibits an indolent yet persistent course, with relative resistance to cytotoxic chemotherapy. Complete surgical resection (R0) remains the strongest prognostic factor (2–4), yet achieving this is often complicated by anatomical extension and functional limitations.

Advanced LGSC with extra-pelvic invasion poses unique challenges that demand a balance between surgical radicality and quality of life (QOL). Although recent guidelines, including the NCCN (2025) and ESMO (2023), emphasize the role of endocrine and targeted therapies in both advanced and recurrent disease (5, 6), initial cytoreductive surgery continues to pivotal role in selected patients. Although extra-pelvic invasion involving major vessels or paraspinal region is generally considered a contraindication for surgery, the indolent biology of LGSC suggests that complete resection—even in anatomically demanding situations—may confer substantial survival advantage compared with high-grade malignancies.

Here, we report a case of FIGO stage IVB LGSC with right inguinal invasion successfully managed through coordinated multidisciplinary surgery. We also introduce a conceptual framework—the “Four Surgical Limits”—to redefine operative boundaries in gynecologic oncology.

## Case presentation

**Figure 1:**
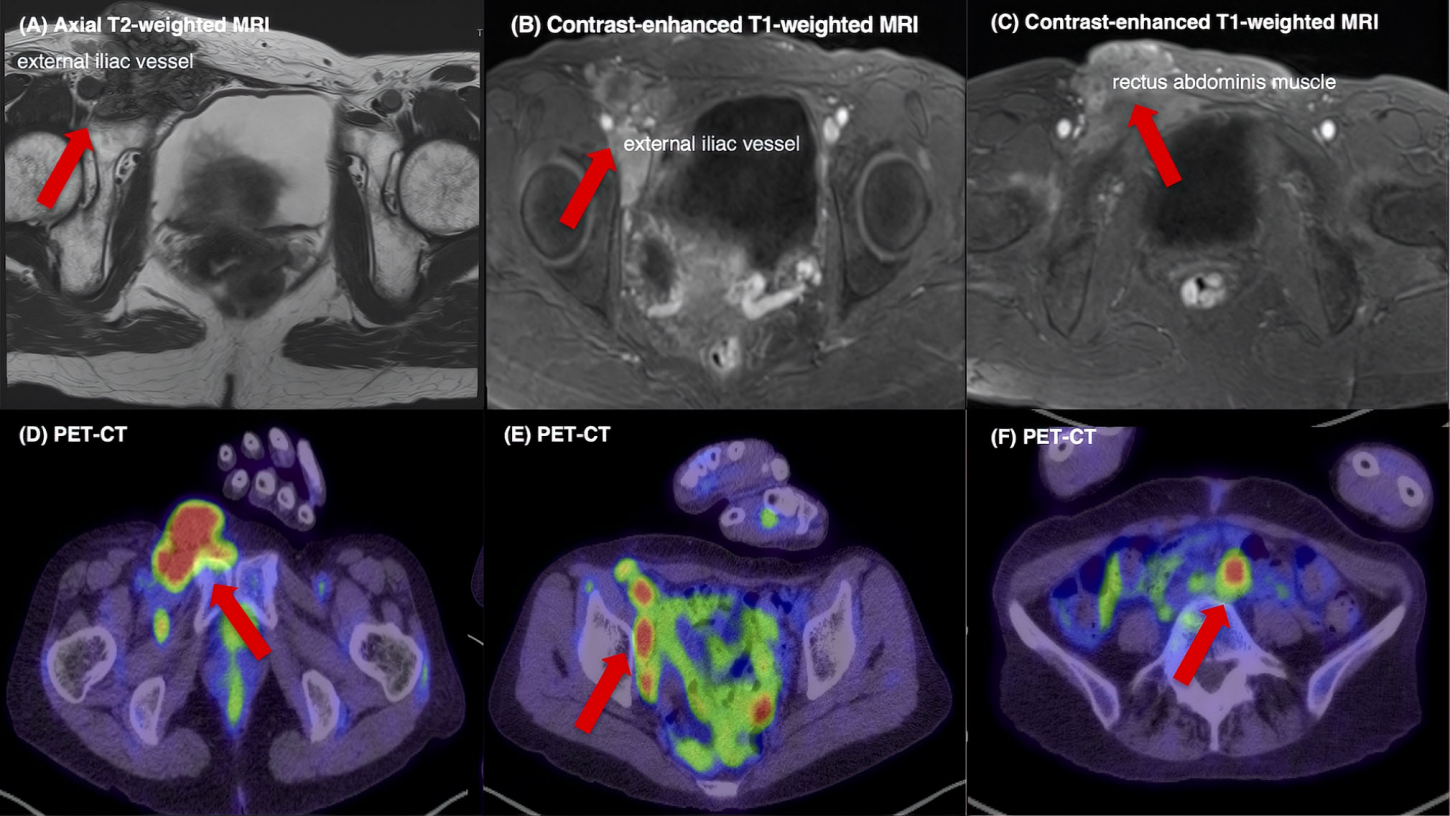
Preoperative MRI and PET-CT findings. **(A)** Axial T2-weighted MRI showing a heterogeneous 9 cm mass in the right inguinal–pelvic region with extension toward the external iliac vessels and infiltration of the lower abdominal wall. **(B)** Contrast-enhanced T1-weighted MRI demonstrating focal involvement and displacement of the external iliac/femoral vein. **(C)** Contrast-enhanced T1-weighted MRI showing linear enhancement along the rectus abdominis muscle, consistent with anterior abdominal wall infiltration. **(D)** PET-CT maximum-intensity projection and axial fusion images showing high FDG uptake in the right inguinal lesion, together with **(E)** multiple FDG-avid pelvic lymph nodes and **(F)** para-aortic lymph nodes, indicating extra-pelvic disease extension consistent with systemic nodal metastases. FDG uptake in the bilateral ovarian tumors was also observed but is not shown.

A 62-year-old woman presented with progressive right inguinal swelling that she first noticed in early May. The mass gradually enlarged, prompting evaluation at a referring hospital. She also reported abdominal distension and persistent abdominal pain that interfered with her daily activities prior to referral. MRI revealed a 9 cm irregular right inguinal mass with heterogeneous internal characteristics and suspected invasion of the femoral vein and lower abdominal wall. In addition, bilateral adnexal tumors were identified, raising concern for an ovarian malignancy with extra-pelvic extension. Transvaginal ultrasound confirmed bilateral complex adnexal masses with preserved mobility. Serologic evaluation demonstrated mildly elevated CA125 (59.8 U/mL), whereas CA19-9, TFPI2, and HE4 were within normal limits. PET-CT revealed high FDG uptake in the bilateral ovarian tumors (left: SUVmax 8.2; right: SUVmax 5.7), the right inguinal lesion (SUVmax 10.2), and multiple pelvic (SUVmax 8.3) and para-aortic lymph nodes (SUVmax 8.94), suggesting systemic nodal involvement without evidence of additional distant metastases (**Figure 1**).

Given the unusual presentation of a large inguinal tumor with concurrent adnexal masses, an ultrasound-guided core-needle biopsy of the inguinal lesion was performed. Histopathology was consistent with low-grade serous carcinoma (PAX8+, WT1+, ER+, p53 wild-type) (**Supplementary Figure 1**). These findings supported the diagnosis of ovarian LGSC with inguinal and nodal metastases, corresponding to a preoperative FIGO stage of IVB.

Following multidisciplinary tumor board discussion, curative-intent resection was selected after a comprehensive review of oncologic, anatomic, functional, and collaborative feasibility. From an oncologic perspective, LGSC is intrinsically resistant to cytotoxic chemotherapy and has limited radiotherapy sensitivity, making meaningful tumor shrinkage with neoadjuvant chemotherapy or upfront targeted agents—including MEK inhibitors—unlikely. Thus, primary debulking surgery (PDS) offered the highest probability of disease control. Anatomically, the absence of ascites and radiologic peritoneal carcinomatosis suggested that complete intra-abdominal cytoreduction was achievable. The major challenge involved the right inguinal mass with suspected vascular invasion.

Orthopedic oncologists confirmed that the inguinal tumor could be resected by wide en bloc resection aiming for negative surgical margins, including partial involvement of the femoral vein, with the feasibility of primary venous repair. Plastic surgeons also confirmed the feasibility of soft-tissue reconstruction using an anterolateral thigh (ALT) flap.

**Figure 2:**
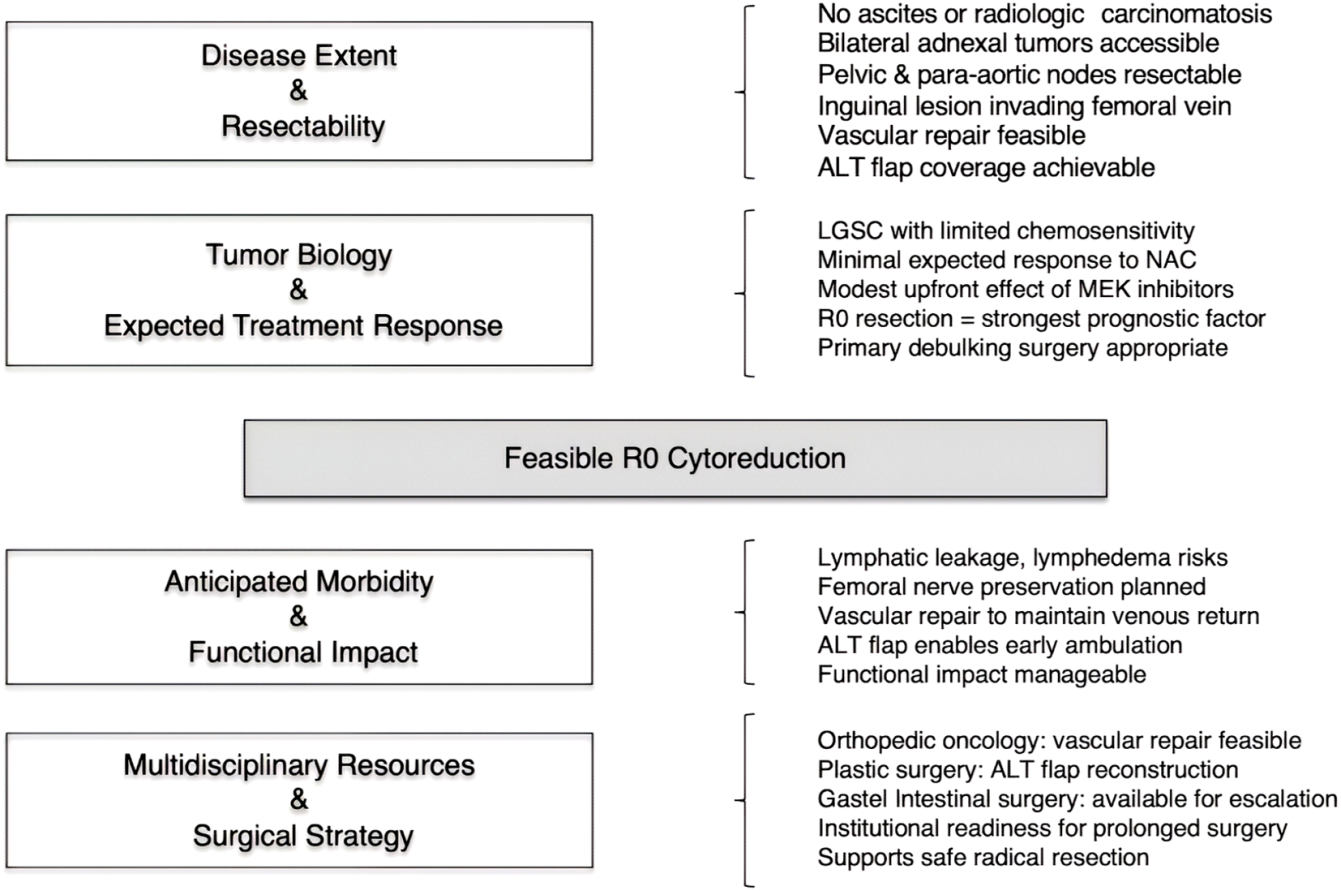
Multidisciplinary feasibility assessment for complete cytoreduction. This framework summarizes the determinants of achieving an R0 cytoreduction in chemotherapy-resistant LGSC by integrating disease extent, tumor biology, expected morbidity, and multidisciplinary resources. When anatomical/oncologic feasibility aligns with functional and reconstructive safety, radical surgery can be justified despite limited systemic treatment response.

Although no formal preoperative consultation with gastrointestinal surgery was obtained due to the absence of radiologic findings suggestive of bowel involvement, the gastrointestinal team remained on standby and agreed to provide intraoperative support should bowel resection become necessary. Potential postoperative morbidities—including lymphedema, lymphatic leakage, and lower-limb sensory or motor deficits—were discussed in detail. The multidisciplinary team concluded that the benefits of complete macroscopic resection outweighed these risks (**Figure 2**).

The surgical procedures included total abdominal hysterectomy, bilateral salpingo-oophorectomy, omentectomy, systematic pelvic and para-aortic lymphadenectomy, and en bloc resection of the right inguinal lesion with partial femoral vein involvement.

**Figure 3:**
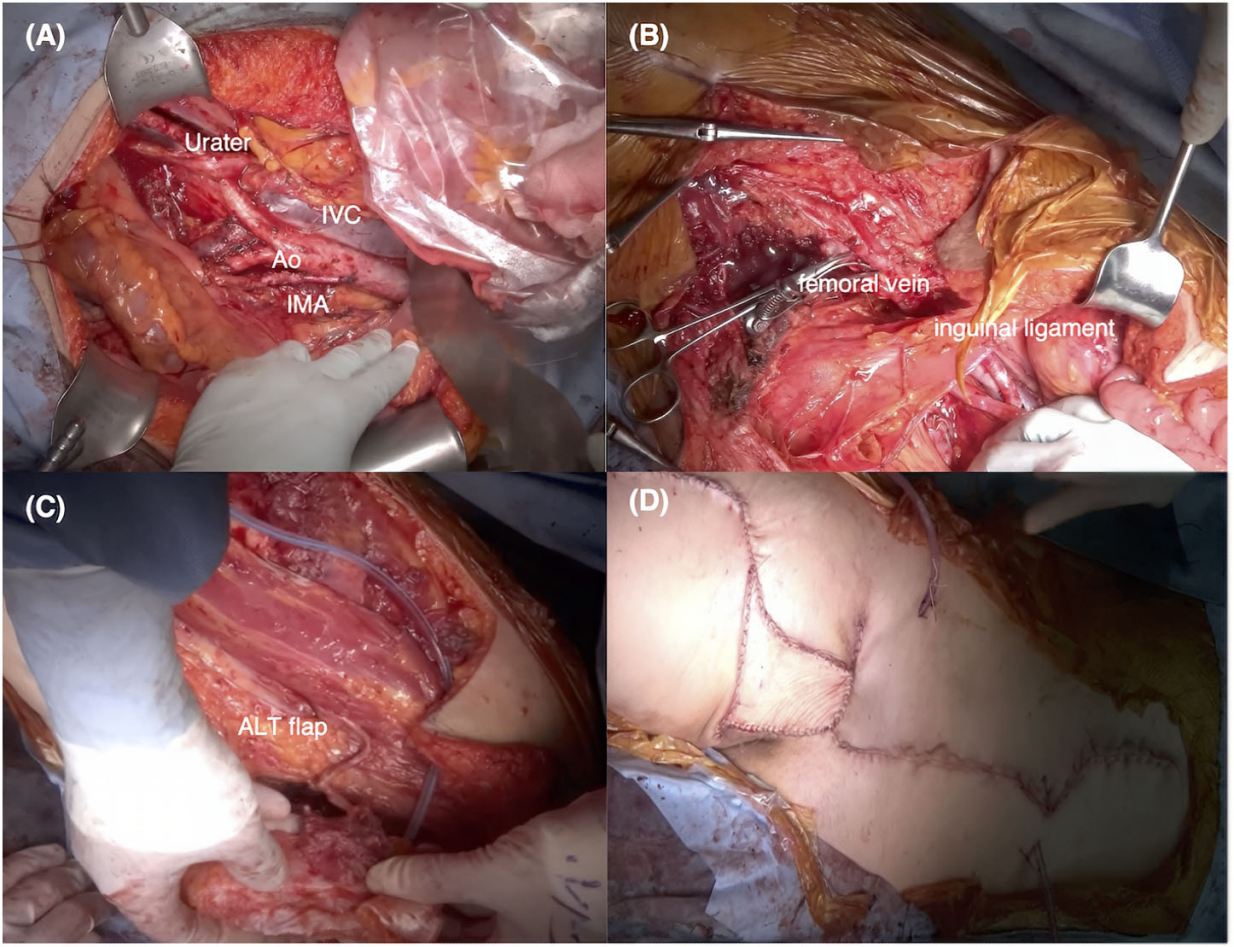
Intraoperative images of the multidisciplinary surgical procedure performed. **(A)** The diagram outlines the extent of en bloc cytoreduction, including total abdominal hysterectomy, bilateral salpingo-oophorectomy, omentectomy, and pelvic and para-aortic lymphadenectomy. Key anatomical landmarks are annotated as follows: IVC, inferior vena cava; Ao, aorta; IMA, inferior mesenteric artery. **(B)** En bloc removal of the right inguinal–abdominal wall lesion with partial femoral vein resection is depicted. **(C, D)** Reconstruction using an ipsilateral anterolateral thigh (ALT) flap is illustrated, highlighting the coordinated contributions of gynecologic oncology, orthopedic oncology, plastic surgery, and gastrointestinal surgery.

Systematic lymphadenectomy was performed because multiple pelvic and para-aortic lymph nodes were diffusely enlarged, making selective or biopsy-based resection insufficient for achieving complete macroscopic disease clearance. The femoral vein was repaired primarily by the orthopedic team. Unexpected peritoneal dissemination involving the transverse colon required partial transverse colectomy with end-to-end anastomosis. The soft-tissue defect was reconstructed using an ipsilateral ALT flap (**Figure 3**). The total operative time was 14 hours 36 minutes, and estimated blood loss was 870 mL.

Postoperative recovery was largely uneventful. From the patient’s perspective, right lower-limb tightness, pain, and sensory disturbance involving the medial thigh and peripatellar region transiently limited daily activities in the early postoperative period. Ambulation with a walker was initiated at 1 week after surgery, progressing to independent walking by 2 weeks and stair climbing by 3 weeks postoperatively. Transient right-lower-limb edema (CTCAE Grade 2) and mild sensory disturbance (Grade 1) were noted but gradually resolved within 4 months. No major complications (Clavien–Dindo ≥ Grade III) occurred. The patient regained full ambulation by 6 weeks postoperatively.

Final pathology confirmed LGSC involving both ovaries, pelvic and para-aortic lymph nodes, and the right inguinal mass, with additional peritoneal dissemination infiltrating the muscular layer of the transverse colon. Based on these findings, the disease was classified as FIGO stage IVB.

Considering the limited sensitivity of LGSC to conventional chemotherapy and the achievement of complete macroscopic resection, adjuvant endocrine therapy with an aromatase inhibitor was recommended as maintenance treatment; however, the patient declined systemic therapy after being informed that the expected benefit in this clinical setting is limited. From the patient’s perspective, she had experienced some difficulty with right lower-limb movement even before treatment but reported a smooth return to daily activities after surgery, resuming work approximately 4 months postoperatively.

Postoperatively, the patient was followed by the gynecologic oncology team every 2–3 months during the first year, including physical examination and serum tumor marker assessment. Given the rarity of LGSC and the advanced disease stage, contrast-enhanced CT was performed at 2 months postoperatively, followed by surveillance CT every 6 months thereafter. At 13 months after surgery, the patient remains alive with no evidence of disease recurrence. Given the indolent yet persistent nature of LGSC, long-term surveillance remains essential. A detailed timeline of the clinical course is summarized in **Supplementary Table 1**.

## Discussion

### Feasibility of R0 resection in chemotherapy-resistant LGSC

This case demonstrates that complete gross resection (R0) can be both feasible and clinically meaningful in chemotherapy-resistant LGSC when performed within a coordinated multidisciplinary framework. LGSC is characterized by intrinsically low sensitivity to cytotoxic chemotherapy compared with high-grade serous carcinoma, and neoadjuvant chemotherapy is generally not recommended as first-line treatment (1, 5, 6). Although endocrine therapy and MEK inhibitors have emerged as systemic options, their upfront effectiveness remains limited; even trametinib, despite being the most promising agent to date, yields modest response rates and is primarily used in the recurrent setting (2). Given these therapeutic constraints, achieving R0 resection represents the single most decisive determinant of long-term outcomes in LGSC and, in many cases, the only intervention capable of meaningfully altering the disease trajectory.

Evidence from tertiary referral centers has further clarified the relationship between surgical extent, morbidity, and survival in advanced ovarian cancer. A large single-center analysis by Aksan et al. (7) comparing standard-radical and ultra-radical primary cytoreductive surgery demonstrated that survival outcomes were driven predominantly by complete cytoreduction and metastatic burden rather than surgical category alone, emphasizing that routine escalation to ultra-radical procedures is not inherently associated with improved prognosis. Similarly, population-based data reported by Falconer et al. (8) showed that although ultra-radical surgery significantly increased R0 resection rates, it did not translate into improved overall survival, underscoring the importance of appropriate patient selection and institutional expertise rather than surgical aggressiveness per se.

In this context, the pursuit of radical cytoreduction must be balanced against preservation of function and quality of life. When implemented within a multidisciplinary surgical strategy integrating gynecologic oncology, vascular, orthopedic, plastic, and gastrointestinal expertise, extensive resection can be performed in a manner that minimizes functional compromise while maintaining postoperative QOL. Our case represents a carefully selected scenario in which tumor biology, anatomical resectability, and comprehensive multidisciplinary support collectively justified an aggressive surgical approach, particularly given the chemotherapy-resistant nature of LGSC.

### Surgical radicality and functional outcomes

A summary of representative studies related to surgical management and multidisciplinary approaches in LGSC is provided in **Supplementary Table 2**. Numerous studies have shown that R0 resection is associated with improved survival outcomes in LGSC (3, 4, 9–12).

However, the value of radical surgery is influenced by tumor biology, dissemination pattern, and the patient’s functional reserve. Matsuo et al. emphasized that although R0 is essential, it is “necessary but not sufficient,” as radical procedures that disregard functional consequences may compromise postoperative QOL and diminish overall benefit (4).

In the present case, despite FIGO stage IVB disease with nodal and extra-pelvic involvement, long-term disease control and preserved ambulation were achieved. This success reflects appropriate oncologic radicality—including en bloc resection involving vascular and bowel structures—combined with deliberate preservation of critical neurovascular pathways and optimized soft-tissue reconstruction.

### A Structured framework incorporating hard and soft surgical limits

**Figure 4:**
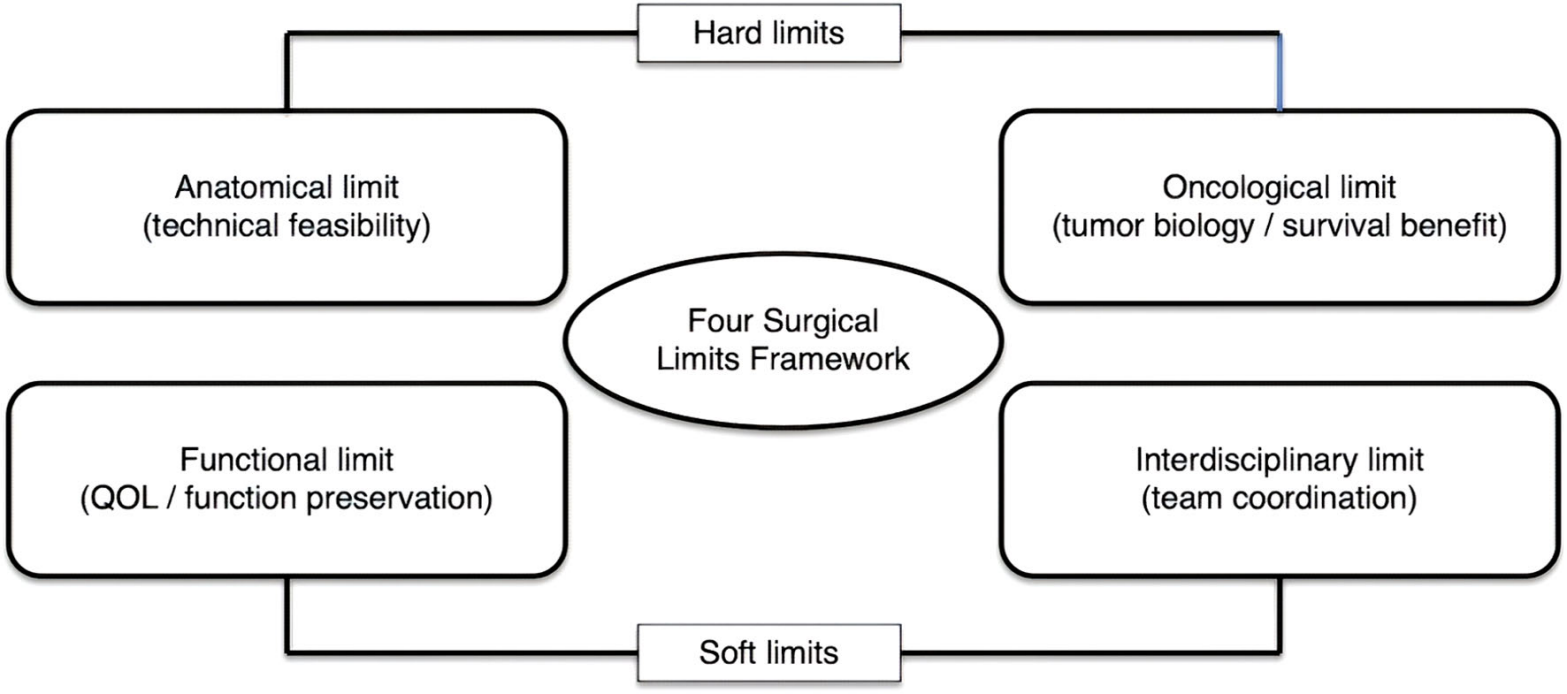
The “Four Surgical Limits” conceptual framework for complex oncologic surgery. A visual model illustrating four domains that guide surgical decision-making: (1) Anatomical limit—defines technical feasibility based on disease distribution, organ invasion, and reconstructive potential. (2) Oncological limit—reflects tumor biology, sensitivity, and expected survival benefit from radical surgery. (3) Functional limit—represents acceptable morbidity thresholds, QOL preservation, and rehabilitation potential. (4) Interdisciplinary limit—captures institutional readiness, cross-specialty coordination, and intraoperative adaptive capacity. The framework emphasizes that R0 resection is meaningful only when both hard limits (anatomical/oncological) and soft limits (functional/interdisciplinary) are respected.

Given the complexity of cytoreductive surgery in advanced LGSC, we applied a structured decision-making framework comprising hard limits (anatomical and oncological) and soft limits (functional and interdisciplinary) (**Figure 4**).

Hard limits determine whether R0 resection is theoretically achievable, whereas soft limits determine whether it is practically and ethically appropriate. This balance is particularly relevant in LGSC, where systemic therapies often provide limited tumor reduction (1, 9, 11, 12).

### Hard limits

#### Anatomical limit

Defined by disease extent, organ invasion, and the feasibility of safe resection and reconstruction. R0 resection should not be pursued when these are not achievable.

#### Oncological limit

Determined by tumor biology and expected survival benefit. LGSC’s poor chemosensitivity expands the oncological limit toward more aggressive cytoreduction (1, 2, 9–12).

### Soft limits

#### Functional limit

Reflects the acceptable threshold of physiological loss and expected morbidity. Functional limits can be expanded through nerve-sparing techniques, reconstructive strategies, and shared decision-making.

#### Interdisciplinary limit

Determined by the coordination, experience, and readiness of involved specialties. Complex surgery requires seamless collaboration across gynecologic, orthopedic, plastic, and gastrointestinal teams.

This conceptual framework is consistent with previously reported experiences from tertiary referral centers (7, 8). Comparative studies evaluating standard-radical versus ultra-radical cytoreductive surgery have demonstrated that surgical feasibility and survival benefit are primarily determined by the ability to achieve complete cytoreduction within acceptable morbidity, rather than by surgical aggressiveness alone. In particular, reports by Aksan et al. and Falconer et al. emphasize that appropriate patient selection, institutional expertise, and multidisciplinary infrastructure are critical for safely expanding the boundaries of surgical resectability. These findings support the relevance of our Four Surgical Limits framework by positioning anatomical feasibility, acceptable functional compromise, and interdisciplinary readiness as interdependent factors that define the practical limits of aggressive cytoreduction in real-world tertiary-care settings.

### Application of hard and soft limits to the present case

#### Anatomical limit

Preoperative imaging revealed bilateral adnexal disease, pelvic and para-aortic lymph node metastases, and a right inguinal mass invading the femoral vein, but no ascites or radiologic peritoneal carcinomatosis—all findings consistent with feasible complete cytoreduction (3, 9–12). The inguinal mass was considered resectable with vascular reconstruction.

#### Oncological limit

Given the well-documented chemotherapy-resistance of LGSC and the absence of strong evidence supporting neoadjuvant chemotherapy (1, 9, 11), primary debulking surgery was favored. Endocrine therapy and MEK inhibitors were considered adjunctive rather than primary options (1, 2).

#### Functional limit

Anticipated morbidities included lymphedema, lymphatic leakage, and potential sensory or motor deficits. Femoral nerve preservation and use of an ipsilateral ALT flap minimized long-term impairment, resulting in only transient Grade 1–2 complications.

#### Interdisciplinary limit

Successful oncologic resection required coordinated involvement of gynecologic oncology, orthopedic oncology, plastic surgery, and gastrointestinal surgery. The institution’s capacity for rapid intraoperative escalation of expertise expanded the interdisciplinary limit, enabling safe management of unexpected transverse colon invasion.

### Clinical implications for advanced LGSC

This case illustrates how a dual-layered evaluation—hard limits defining feasibility and soft limits ensuring appropriateness—can operationalize the principle that “R0 is necessary but not sufficient” in advanced LGSC (4).

In chemotherapy-resistant disease, meaningful outcomes may be achieved when R0 resection is pursued within anatomically and oncologically justified boundaries while preserving postoperative function through tailored surgical planning and strong interdisciplinary collaboration.

Our patient, who underwent extensive R0 resection, experienced minimal morbidity, declined endocrine therapy, and remains disease-free at 13 months—an outcome consistent with emerging evidence supporting surgery-centered strategies in this population (1, 3, 9–12). Although long-term prognosis must be interpreted with caution, the achievement of R0 resection provided immediate clinical benefits, including marked improvement in quality of life through the resolution of inguinal edema and pain, alongside short-term disease control. These observations underscore the potential value of thoughtfully executed multidisciplinary cytoreduction even in anatomically complex presentations of LGSC.

Further prospective studies are warranted to validate this framework and clarify how institutional factors, surgical expertise, and multidisciplinary infrastructure influence both functional and interdisciplinary limits (1, 13).

### Patient perspective

“Before surgery, I experienced abdominal distension and persistent abdominal pain that limited my daily activities, as well as discomfort and pain in my right lower limb. After the procedure, my abdominal symptoms resolved, and although I temporarily experienced tightness and sensory changes in my right leg, these gradually improved with rehabilitation, allowing me to return to my normal daily life. Throughout the treatment process, I felt reassured by the close collaboration of multiple specialists, who provided coordinated and comprehensive care.”

## Conclusion

Multidisciplinary collaboration can transcend traditional surgical and oncologic boundaries in advanced gynecologic malignancies. This case demonstrates that coordinated operative planning enables both radical resection and functional preservation in chemotherapy-resistant LGSC, underscoring the value of a framework-based approach to defining surgical limits.

## Data Availability

The original contributions presented in the study are included in the article/**Supplementary Material**. Further inquiries can be directed to the corresponding author.

## Supplementary material

Supplementary Figure 1 Histopathological and immunohistochemical findings of the right inguinal lesion. **(A)** Hematoxylin and eosin (H&E) staining shows papillary and micropapillary proliferation of atypical cuboidal to columnar epithelial cells with mild nuclear atypia, consistent with low-grade serous carcinoma. **(B–E)** Immunohistochemical staining demonstrates diffuse nuclear positivity for PAX8, WT1, and ER, with a wild-type (heterogeneous) staining pattern for p53.
