# Supplementary figures and images for: Case Report: Multidisciplinary approach for complete resection of primary advanced low-grade serous ovarian carcinoma involving the iliac vessels and paraspinal region
Source: Front Oncol. 2026 Feb 25;16:1762009. doi: 10.3389/fonc.2026.1762009 (PMC12975547; doi:10.3389/fonc.2026.1762009)

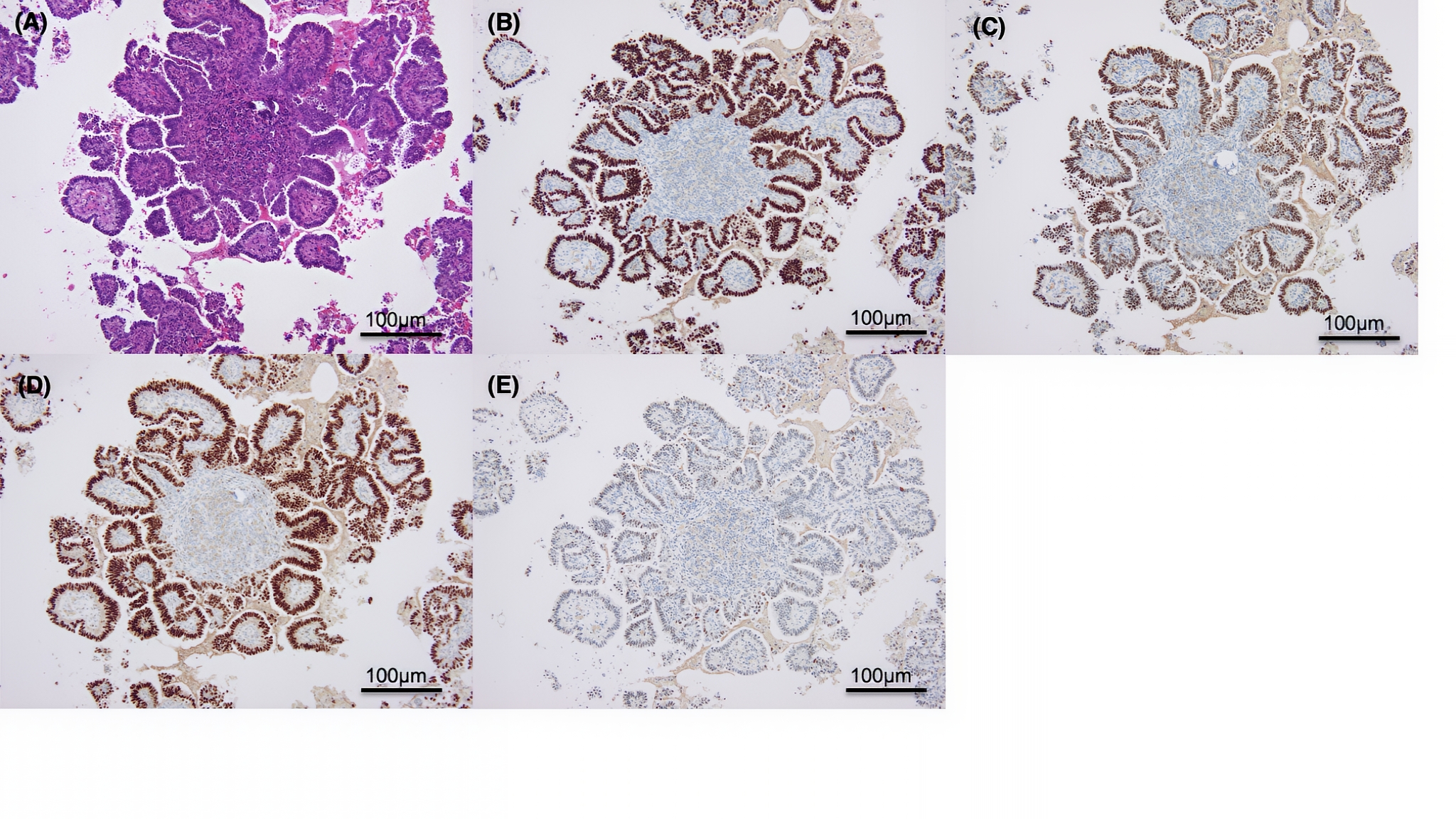

Supplement: Supplementary Figure 1 — Histopathological and immunohistochemical findings of the right inguinal lesion. (A) Hematoxylin and eosin (H&E) staining shows papillary and micropapillary proliferation of atypical cuboidal to columnar epithelial cells with mild nuclear atypia, consistent with low-grade serous carcinoma. (B–E) Immunohistochemical staining demonstrates diffuse nuclear positivity for PAX8, WT1, and ER, with a wild-type (heterogeneous) staining pattern for p53. [file Image1.jpeg]
